# Association between fractures and health status among independent older adults: insights from a suburban cohort in Japan

**DOI:** 10.1186/s12877-025-05876-y

**Published:** 2025-04-10

**Authors:** Shuzo Tsujimura, Takehiro Michikawa, Akira Tsuzuki, Takashi Kuroiwa, Soya Kawabata, Yusuke Kawano, Mitsuhiro Morita, Kazue Hayakawa, Shinjiro Kaneko, Hajime Takechi, Nobuyuki Fujita

**Affiliations:** 1 Department of Orthopaedic Surgery, School of Medicine, Fujita Health University, Aichi, Japan; 2 Department of Environmental and Occupational Health, School of Medicine, Toho University, Tokyo, Japan; 3 Faculty of Rehabilitation, School of Health Science, Fujita Health University, Aichi, Japan; 4 Department of Spine and Spinal Cord Surgery, School of Medicine, Fujita Health University, Aichi, Japan; 5 Department of Geriatrics and Cognitive Disorders, School of Medicine, Fujita Health University, Aichi, Japan

**Keywords:** Fracture, Older well-aging adult, Community dwelling, Subjective geriatric complaint, Dysphagia

## Abstract

**Background:**

Fractures are a significant health concern for older adults, affecting their activities of daily living (ADL), physical function, and mental well-being, and contributing to the need for long-term care. However, the factors associated with fractures among independent older adults remain unclear. This study aimed to examine the association between fractures and the health status of independent older adults in a representative suburban city in Japan using data from a survey of community-dwelling residents and to identify factors associated with fractures.

**Methods:**

A total of 15,853 survey questionnaires were mailed, and 11,346 valid responses were received, resulting in a response rate of 71.6%. The survey included questions on ADL, physical function, mental health, memory, medical conditions, and subjective complaints. For fractures, participants were asked the following question: “How many times have you experienced fractures since the age of 65?”. Data were analyzed using Poisson regression models adjusted for age, sex, body mass index, family structure, and smoking history.

**Results:**

Among the respondents, 15% reported fractures after age of 65 years. A decline in ADL, physical function, mental health, and memory were significantly associated with increased fracture frequency. Under medical conditions, the prevalence of depression (*p* for trend = 0.042), respiratory diseases (*p* for trend = 0.001), and ophthalmologic conditions (*p* for trend = 0.002) increased significantly with fracture number. Most subjective complaints were significantly associated with fracture number, with dysphagia demonstrating the strongest association.

**Conclusions:**

This study utilized a relatively large and highly representative sample of community-dwelling residents to identify factors associated with fractures in independent older adults. Even in independent older adults who were not certified as requiring long-term care, fractures were significantly associated with a decline in multiple health domains. These findings provide valuable insights that can inform efforts to promote healthy aging and reduce care dependency.

**Clinical trial:**

Not applicable.

## Introduction

In a rapidly aging society, maintaining health span and improving the quality of life for older adults have become important challenges. Among the various health issues affecting older individuals, fragility fractures are a major concern, significantly contributing to a decline in activities of daily living (ADL), progression to a care-dependent state, and increase in mortality risk [1, 2]. Fragility fractures caused by falls are highly prevalent in older adults, underscoring the need for prevention and intervention strategies [3]. The causes of fragility fractures in older adults are closely linked to osteoporosis and increased fall risk [4]. Fatigue, dizziness, balance instability, and decline in vision and hearing precipitate falls, consequently causing fragility fractures [5–7]. Moreover, older adults often have multiple medical conditions, such as hypertension, diabetes, and cardiovascular disease, and are frequently prescribed medications that can induce side effects, further compounding the risk of falls and fragility fractures [8].

While it is crucial to understand the risk factors leading to fragility fractures, it is equally important to investigate the consequences of fragility fractures on the health and well-being of older adults. Fragility fractures can exacerbate subjective complaints, contribute to functional decline, and negatively impact mental health by causing depressive symptoms, loss of self-confidence, and social isolation, potentially increasing the risk of dementia [9–12]. The long-term effects of fragility fractures on physical function and overall health remain incompletely understood, necessitating further research.

In Japan, a public long-term care insurance system was introduced in 2000 to address the challenges of an aging society [13]. This system enables older adults who were certified as requiring care to access a wide range of support services, including in-home care and facility-based care. Although the system focuses on providing support to those with significant care needs, it offers preventive care services aimed at older adults with mild care needs, classified as requiring support. According to the 2022 Comprehensive Survey of Living Conditions conducted by Ministry of Health, Labour and Welfare, Japan, fractures and falls accounted for 13.0% of the primary causes of long-term care certification in Japan [14], making them the third most common cause, following dementia and cerebrovascular diseases. However, the prevalence of fractures among “independent older adults” who have not been certified as requiring long-term care is unclear. Furthermore, to what extent fractures are associated with ADL, physical function, depression, or cognitive function among independent older adults requires further investigation. Similarly, the relationship between fractures and other medical conditions or subjective complaints among independent older adults needs to be determined.

Given the high likelihood that independent older adults may require long-term care certification in the future, clarifying the prevalence of fractures and the health status of in this population is essential. Understanding these situations associated with fractures in this population will provide fundamental data for establishing effective preventive measures to extend healthy life expectancy. Thus, this study aimed to examine the association between fractures and the health status of independent older adults in a representative suburban city in Japan using cross-sectional data from a survey of community-dwelling residents. By elucidating the relationship between past fractures and current health status, we seek to enhance the understanding of how fractures affect the lives of older adults and contribute to the development of strategies for better management and support.

## Materials and methods

### Survey on the living conditions of older adults

**Fig. 1:**
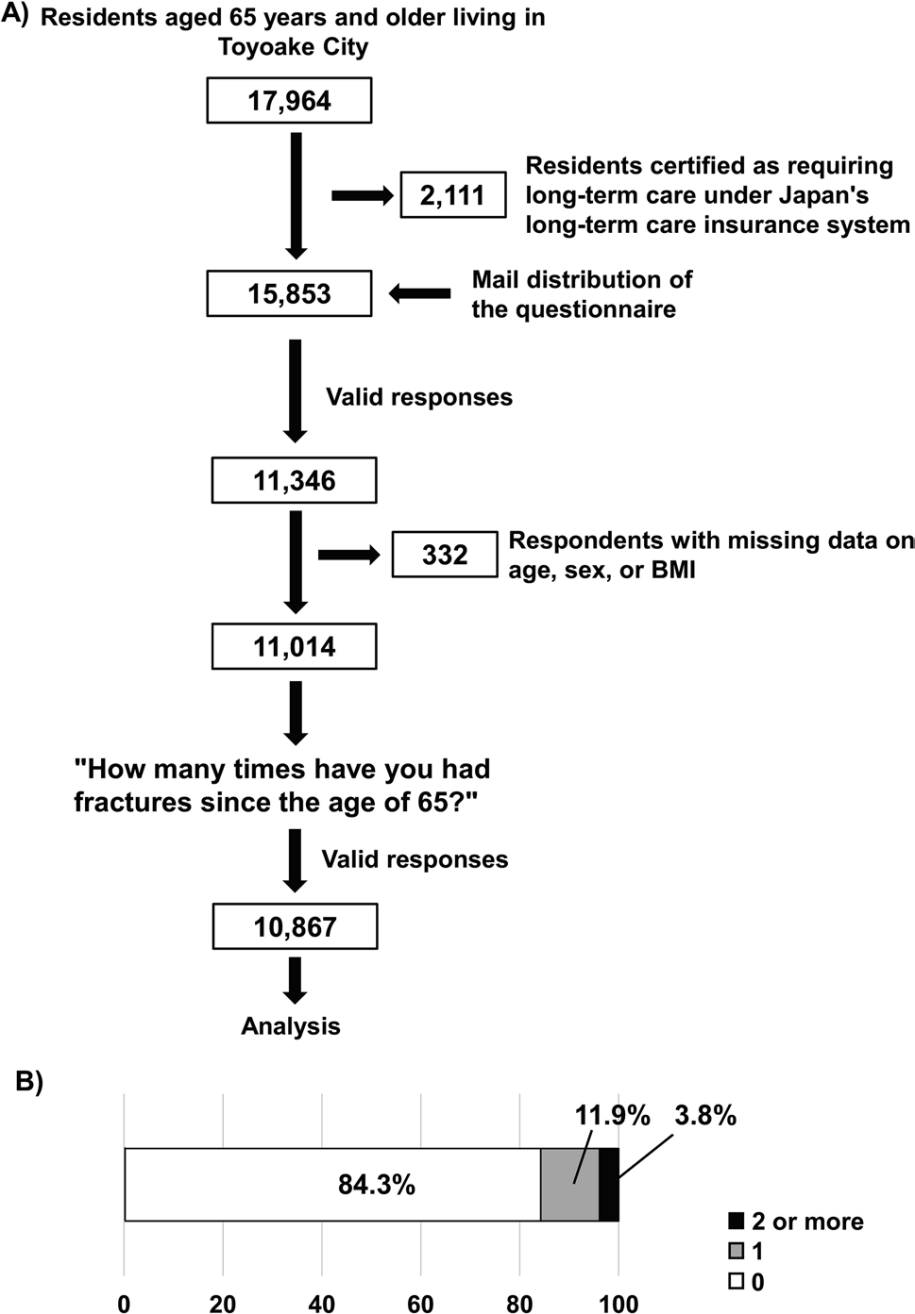
Survey of community-dwelling residents in the city of Toyoake. (**A**) Response flowchart to the survey questionnaire for residents aged ≥ 65 years. (**B**) Fracture number since the age of 65

This survey was conducted by the city of Toyoake, in suburban Aichi Prefecture, Japan, from December 2022 to January 2023, to assess the living conditions, health risks, and social participation status of older adults before the onset of care dependency. Among the 17,964 residents aged ≥ 65 years living in Toyoake City, 2,111 were certified as requiring long-term care under Japan’s long-term care insurance system, whereas 15,853 were not (Fig. 1A). The latter group, namely independent older adults, was included as the target population for this study. A total of 15,853 survey questionnaires were distributed, and 11,346 valid responses were received, resulting in a response rate of 71.6% (Fig. 1A). Of these, 332 respondents with missing data on age, sex, or body mass index (BMI) were excluded, resulting in 11,014 valid responses (Fig. 1A).

### Ethics approval

This study was approved by the ethics committees of our institution (HM24-188). Written informed consent was obtained from all participants. The study adhered to the guidelines of the Declaration of Helsinki.

### Questionnaire

The questionnaire consisted of items covering six domains as follows: personal and family circumstances, physical activity, daily living, community activities and mutual support, dietary habits, and health status [15]. These domains were designed to be relevant and accessible for responses from community residents. From this questionnaire, baseline characteristics, including age, sex, BMI, family composition, and smoking history, were initially extracted.

Based on a previous study [16], the following items were selected for questions related to physical function: (1) “Do you normally climb stairs without using a handrail or wall for support?,” (2) “Do you normally stand up from a chair without any aid?,” (3) “Do you normally walk continuously for 15 minutes?,” (4) “Have you experienced any falls in the past year?,” and (5) “Do you have a fear of falling while walking?.” We defined reduced physical function as the condition in which three or more of these five questions are applicable.

In accordance with assessments of instrumental ADL (IADL) in older adults for daily living [17], the following items were selected: (1) “Do you go out by bus or train by yourself?,” (2) “Do you do your own grocery and daily necessities shopping?,” (3) “Do you prepare your own meals?,” and (4) “Do you manage deposits and withdrawals from your bank accounts on your own?.” For responses, when the options included “able and do,” “able but do not,” and “unable,” the first two options were considered “yes” and the third option, “unable,” was considered “no.”

The following items were selected for depression: (1) “Have you felt down, depressed, or hopeless in the past month?” and (2) “Have you been bothered by little interest or pleasure in doing things in the past month?” [18].

The following items were selected for memory: (1) “Do you find yourself not knowing today’s date?” and (2) “Do your family or friends point out your memory loss?” [16].

For current medical conditions, the presence or absence of the following conditions was assessed, excluding musculoskeletal diseases and trauma related to fractures: hypertension, stroke, heart disease, diabetes, hyperlipidemia, respiratory disease, gastrointestinal disease, renal/urinary tract disease, malignant tumors, hematologic and immune diseases, depression, dementia, Parkinson’s disease, ophthalmologic disease, and otolaryngologic disease.

For subjective geriatric complaints, the presence of the following symptoms was examined: dizziness, urination disorder, defecation disorder, insomnia, low back pain, arthralgia, vision impairment, presbycusis, appetite loss, cough/phlegm, dyspnea, dysphagia, easy fatigability, feeling depressed, forgetfulness, headache, and edema [15]. These lists were created according to existing lists of geriatric syndromes and related publications [19–23]. Symptoms and conditions classified as part of a discrete disease, such as dementia, were excluded from the list. Those often accompanied by severe illnesses not observed in community-dwelling older people living independently, such as delirium or pressure ulcers, were excluded from the list. Multiple responses were allowed for medical conditions and subjective geriatric complaints.

### Fracture

For fractures, the following question were prepared: “How many times have you had fractures since the age of 65?” with five response options: 0, 1, 2, 3, or > 3, and “Do not remember well.”

### Statistical analysis

Fracture number was categorized into three groups (0, 1, and ≥ 2) because a few participants answered ≥ 3. Baseline characteristics according to fracture frequency groups were compared using Pearson’s chi square test (Table 1). We defined the number of fractures as explanatory variable and the health status of independent older adults, including current medical conditions, physical function, instrumental activities of daily living, depression, memory, and subjective geriatric complaints, as outcome variables. Then, we constructed a Poisson regression model that included several potential confounding factors, such as age, sex, BMI, family structures, and smoking habits, and estimated prevalence ratios. A linear trend was tested by assigning ordinal values (1–3) for the fracture frequency groups. Statistical analyses were conducted using SPSS software (version 29.0; IBM Inc., Armonk, NY, USA) and STATA16 software (Stata Corporation, College Station, TX, USA), with *p*-values of < 0.05 indicating a statistically significant difference.

**Table 1:** Distribution of groups for each baseline characteristic based on the number of fractures (*n* = 10,867)

		Total number	The number of fractures			
			0	1	2 or more	*P* value
Age (years)	65–74	5,092	4594 (90.2%)	427 (8.4%)	81 (1.4%)	< 0.001
	75–84	4,657	3759 (80.7%)	663 (14.2%)	235 (5.0%)	
	85 or more	1,118	802 (71.8%)	205 (18.3%)	111 (9.9%)	
Sex	Males	5,065	4581 (90.4%)	399 (7.9%)	85 (1.7%)	< 0.001
	Females	5,802	4574 (78.8%)	896 (15.4%)	332 (5.7%)	
Body mass index (kg/m^2^)	< 18.5	934	749 (80.2%)	128 (13.7%)	57 (6.1%)	< 0.001
	18.5–24.9	7,632	6439 (84.4%)	907 (11.9%)	286 (3.7%)	
	≥ 25.0	2,301	1967 (85.5%)	260 (11.3%)	74 (3.2%)	
Family structures	Living alone	1,763	1398 (79.3%)	259 (14.7%)	106 (6.0%)	< 0.001
	Living as a couple	5,073	4387 (86.5%)	530 (10.4%)	156 (3.1%)	
	Living in a multi-generational household	2,342	1934 (82.6%)	313 (13.4%)	95 (4.1%)	
	Others	1,522	1302 (85.5%)	170 (11.2%)	50 (3.3%)	
Smoking habits	Current smokers	945	847 (89.6%)	78 (8.3%)	20 (2.1%)	< 0.001
	Past smokers	3,179	2846 (89.5%)	268 (8.4%)	65 (2.0%)	
	Never	6,607	5358 (81.1%)	925 (14.0%)	324 (4.9%)	

## Results

10,928 participants responded to the question “How many times have you had fractures since the age of 65?” (Fig. 1A). In total, 61 answered “Do not remember well,” leaving 10,867 valid responses (Fig. 1A). Of these, 3.8% reported ≥ 2 fractures, 11.9% reported 1 fracture, and 84.3% reported no fractures (Fig. 1B).

Table 1 shows the baseline characteristics of the 10,867 participants who provided valid responses regarding fracture number. Comparisons of baseline characteristics across fracture frequency groups showed significant differences in all items (Table 1).

Table 2 shows the prevalence of current medical conditions based on fracture number. Among the 15 current medical conditions, even after adjustment for age, sex, BMI, family structure, and smoking habits, respiratory disease (*p* for trend = 0.001), depression (*p* for trend = 0.042), and ophthalmologic disease (*p* for trend = 0.002) showed a significant positive trend, with an increase in prevalence with fracture number (Table 2).

**Table 2:** Prevalence of current medical conditions based on the number of fractures (*n* = 10,867)

Outcome		No. of fractures			*p* for trend**
		0	1	2 or more	
No. of participants		9,155	1,295	417	
Hypertension	Prevalence of “yes” (%)	43.8	47.8	47.7	
	Adjusted PR (95% CI)*	Ref	1.04 (0.98–1.11)	1.03 (0.92–1.14)	0.246
Stroke	Prevalence of “yes” (%)	3.5	3.9	3.4	
	Adjusted PR (95% CI)*	Ref	1.20 (0.89–1.60)	1.03 (0.61–1.77)	0.406
Heart disease	Prevalence of “yes” (%)	10.0	10.9	12.7	
	Adjusted PR (95% CI)*	Ref	1.06 (0.89–1.26)	1.15 (0.89–1.51)	0.233
Diabetes	Prevalence of “yes” (%)	14.1	14,8	13.0	
	Adjusted PR (95% CI)*	Ref	1.14 (0.99–1.32)	1.04 (0.80–1.35)	0.173
Hyperlipidemia	Prevalence of “yes” (%)	18.1	19.0	17.8	
	Adjusted PR (95% CI)*	Ref	1.03 (0.91–1.16)	0.99 (0.80–1.23)	0.834
Respiratory disease	Prevalence of “yes” (%)	4.9	6.3	7.7	
	Adjusted PR (95% CI)*	Ref	1.32 (1.04–1.67)	1.66 (1.17–2.36)	0.001
Gastrointestinal disease	Prevalence of “yes” (%)	7.3	8.8	11.8	
	Adjusted PR (95% CI)*	Ref	1.11 (0.91–1.34)	1.26 (0.94–1.70)	0.086
Renal/urinary tract disease	Prevalence of “yes” (%)	11.5	9.5	8.9	
	Adjusted PR (95% CI)*	Ref	1.02 (0.86–1.21)	1.07 (0.78–1.47)	0.659
Malignant tumor	Prevalence of “yes” (%)	3.8	4.4	3.1	
	Adjusted PR (95% CI)*	Ref	1.28 (0.96–1.69)	0.85 (0.48–1.51)	0.518
Hematologic and immune disease	Prevalence of “yes” (%)	4.6	4.8	5.0	
	Adjusted PR (95% CI)*	Ref	0.97 (0.74–1.27)	1.04 (0.67–1.62)	0.987
Depression	Prevalence of “yes” (%)	1.1	1.7	1.7	
	Adjusted PR (95% CI)*	Ref	1.64 (1.02–2.62)	1.61 (0.71–3.64)	0.042
Dementia	Prevalence of “yes” (%)	0.4	0.9	0.7	
	Adjusted PR (95% CI)*	Ref	1.93 (0.97–3.83)	0.72 (0.16–3.17)	0.475
Parkinson	Prevalence of “yes” (%)	0.4	0.5	1.2	
	Adjusted PR (95% CI)*	Ref	1.14 (0.49–2.65)	2.85 (1.03–7.87)	0.094
Ophthalmologic disease	Prevalence of “yes” (%)	15.8	20.4	26.4	
	Adjusted PR (95% CI)*	Ref	1.11 (0.99–1.26)	1.28 (1.08–1.52)	0.002
Otolaryngology disease	Prevalence of “yes” (%)	3.5	3.5	5.8	
	Adjusted PR (95% CI)*	Ref	0.88 (0.64–1.20)	1.26 (0.83–1.91)	0.713

CI: confidence interval, PR: prevalence ratio

*We applied a Poisson regression model and adjusted for age, sex, body mass index, family structures and smoking habits

**A linear trend was tested by assigning ordinal values for fracture frequency groups

Table 3 shows the prevalence of “No” or“Yes” to questions on physical function, IADL, depression, and memory based on fracture number. For all items, even after adjustment for age, sex, BMI, family structure, and smoking habits, prevalence increased significantly with fracture number (Table 3).

**Table 3:** Prevalence of “no” or “yes” to questions on physical function, instrumental activities of daily living, depression, and memory based on the number of fractures

Outcome		No. of fractures			*p* for trend**
		0	1	2 or more	
Physical function	**1) Do you normally climb stairs without using a handrail or wall for support?**				
	No of participants	9,091	1,283	410	
	Prevalence of “No” (%)	12.3	26.4	44.9	
	Adjusted PR (95% CI)*	Ref	1.54 (1.38–1.71)	2.02 (1.77–2.31)	< 0.001
	**2) Do you normally stand up from a chair without any aids?**				
	No of participants	9,113	1,289	413	
	Prevalence of “No” (%)	7.8	16.8	43.0	
	Adjusted PR (95% CI)*	Ref	1.52 (1.32–1.75)	2.17 (1.82–2.59)	< 0.001
	**3) Do you normally walk continuously for 15 min?**				
	No of participants	9,091	1,282	411	
	Prevalence of “No” (%)	5.1	12.0	20.4	
	Adjusted PR (95% CI)*	Ref	1.72 (1.44–2.05)	2.20 (1.75–2.75)	< 0.001
	**4) Have you experienced any falls in the past year?**				
	No of participants	9,113	1,284	411	
	Prevalence of “Yes” (%)	21.1	40.4	54.3	
	Adjusted PR (95% CI)*	Ref	1.79 (1.65–1.94)	2.24 (2.02–2.48)	< 0.001
	**5) Do you have a fear of falling while walking?**				
	No of participants	9,095	1,280	412	
	Prevalence of “Yes” (%)	44.2	67.9	81.8	
	Adjusted PR (95% CI)*	Ref	1.33 (1.27–1.39)	1.42 (1.35–1.51)	< 0.001
	**6) Do three or more of the above five questions apply?**				
	No of participants	8,972	1,252	398	
	Prevalence of “Yes” (%)	9.3	22.9	39.7	
	Adjusted PR (95% CI)*	Ref	1.75 (1.55–1.97)	2.27 (1.95–2.64)	< 0.001
ADL	**1) Do you go out by bus or train by yourself?**				
	No of participants	9.079	1,276	412	
	Prevalence of “No” (%)	4.8	11.8	24.5	
	Adjusted PR (95% CI)*	Ref	1.55 (1.30–1.86)	2.23 (1.83–2.72)	< 0.001
	**2) Do you do your own grocery and daily necessities shopping?**				
	No of participants	9.087	1,278	405	
	Prevalence of “No” (%)	3.0	6.7	11.9	
	Adjusted PR (95% CI)*	Ref	1.61 (1.27–2.04)	2.14 (1.60–2.88)	< 0.001
	**3) Do you prepare your own meals?**				
	No of participants	9,092	1,277	407	
	Prevalence of “No” (%)	8.7	7.4	11.1	
	Adjusted PR (95% CI)*	Ref	1.07 (0.88–1.31)	1.81 (1.37–2.40)	0.001
	**4) Do you manage deposits and withdrawals from your bank accounts on your own?**				
	No of participants	9,100	1,278	412	
	Prevalence of “No” (%)	3.9	4.3	7.8	
	Adjusted PR (95% CI)*	Ref	1.06 (0.81–1.40)	1.79 (1.28–2.51)	0.006
Depression	**1) Have you felt down or depressed or hopeless in the past month?**				
	No of participants	9,011	1,255	409	
	Prevalence of “Yes” (%)	31.9	40.6	42.8	
	Adjusted PR (95% CI)*	Ref	1.22 (1.14–1.32)	1.23 (1.09–1.39)	< 0.001
	**2) Have you been bothered by little interest or pleasure in doing things in the past month?**				
	No of participants	9,048	1,270	410	
	Prevalence of “Yes” (%)	19.3	23.8	32.9	
	Adjusted PR (95% CI)*	Ref	1.17 (1.05–1.31)	1.51 (1.30–1.76)	0.001
Memory	**1) Do you find yourself not knowing today’s date?**				
	No of participants	9,092	1,281	414	
	Prevalence of “Yes” (%)	19.0	22.1	25.4	
	Adjusted PR (95% CI)*	Ref	1.10 (0.98–1.23)	1.14 (0.96–1.36)	0.006
	**2) Do your family or your friends point out your memory loss?**				
	No of participants	9,088	1,284	415	
	Prevalence of “Yes” (%)	11.2	14.9	21.9	
	Adjusted PR (95% CI)*	Ref	1.25 (1.08–1.45)	1.70 (1.40–2.08)	< 0.001

CI: confidence interval, PR: prevalence ratio

*We applied a Poisson regression model and adjusted for age, sex, body mass index, family structures and smoking habits

**A linear trend was tested by assigning ordinal values for fracture frequency groups

Finally, the prevalence of subjective geriatric complaints based on fracture number was analyzed (Table 4). For all complaints, excluding appetite loss (*p* for trend = 0.911) and headache (*p* for trend = 0.056), prevalence increased significantly with fracture number. Among the complaints, a clear dose–response relationship was observed for dysphagia. Compared with the no fracture group, the prevalence ratios of dysphagia were 1.34 (95% confidence interval = 1.09–1.65) for 1 fracture group and 2.24 (1.72–2.91) for ≥ 2 fracture groups.

**Table 4:** Prevalence of subjective geriatric complaints based on the number of fractures (*n* = 10,867)

Outcome		No. of fractures			*p* for trend**
		0	1	2 or more	
No. of participants		9,155	1,295	417	
Dizziness	Prevalence of “yes” (%)	13.5	19.7	30.2	
	Adjusted PR (95% CI)*	Ref	1.27 (1.13–1.44)	1.73 (1.47–2.03)	< 0.001
Urination disorder	Prevalence of “yes” (%)	21.9	25.8	32.6	
	Adjusted PR (95% CI)*	Ref	1.20 (1.09–1.33)	1.47 (1.26–1.71)	< 0.001
Defecation disorder	Prevalence of “yes” (%)	11.0	15.2	17.0	
	Adjusted PR (95% CI)*	Ref	1.30 (1.13–1.51)	1.23 (0.98–1.55)	0.001
Insomnia	Prevalence of “yes” (%)	12.7	15.4	20.6	
	Adjusted PR (95% CI)*	Ref	1.11 (0.96–1.28)	1.32 (1.07–1.63)	0.006
Low back pain	Prevalence of “yes” (%)	27.4	34.8	43.7	
	Adjusted PR (95% CI)*	Ref	1.24 (1.14–1.35)	1.50 (1.33–1.69)	< 0.001
Arthralgia	Prevalence of “yes” (%)	18.4	22.8	32.1	
	Adjusted PR (95% CI)*	Ref	1.13 (1.01–1.27)	1.51 (1.30–1.76)	< 0.001
Vision impairment	Prevalence of “yes” (%)	25.8	28.7	32.4	
	Adjusted PR (95% CI)*	Ref	1.09 (0.99–1.20)	1.18 (1.02–1.37)	0.008
Hearing loss	Prevalence of “yes” (%)	15.6	19.9	23.5	
	Adjusted PR (95% CI)*	Ref	1.17 (1.03–1.32)	1.21 (1.01–1.45)	0.003
Appetite loss	Prevalence of “yes” (%)	2.0	2.1	3.6	
	Adjusted PR (95% CI)*	Ref	0.86 (0.57–1.31)	1.18 (0.70-2.00)	0.911
Cough/phlegm	Prevalence of “yes” (%)	6.6	8.8	10.1	
	Adjusted PR (95% CI)*	Ref	1.40 (1.15–1.70)	1.57 (1.15–2.13)	< 0.001
Shortness of breath	Prevalence of “yes” (%)	8.2	9.8	13.9	
	Adjusted PR (95% CI)*	Ref	1.16 (0.97–1.39)	1.54 (1.19–1.99)	0.001
Dysphagia	Prevalence of “yes” (%)	5.7	8.0	14.9	
	Adjusted PR (95% CI)*	Ref	1.34 (1.09–1.65)	2.24 (1.72–2.91)	< 0.001
Easily fatigued	Prevalence of “yes” (%)	15.3	16.8	26.6	
	Adjusted PR (95% CI)*	Ref	0.99 (0.86–1.13)	1.44 (1.21–1.71)	0.004
Feeling depressed	Prevalence of “yes” (%)	5.1	6.5	8.9	
	Adjusted PR (95% CI)*	Ref	1.15 (0.92–1.45)	1.44 (1.04–2.01)	0.021
Forgetfulness	Prevalence of “yes” (%)	11.8	18.1	20.6	
	Adjusted PR (95% CI)*	Ref	1.34 (1.17–1.52)	1.31 (1.07–1.60)	< 0.001
Headache	Prevalence of “yes” (%)	3.6	4.3	5.8	
	Adjusted PR (95% CI)*	Ref	1.16 (0.87–1.53)	1.47 (0.97–2.25)	0.056
Edema	Prevalence of “yes” (%)	4.2	6.2	12.00	
	Adjusted PR (95% CI)*	Ref	1.16 (0.92–1.47)	1.83 (1.37–2.45)	< 0.001

CI: confidence interval, PR: prevalence ratio

*We applied a Poisson regression model and adjusted for age, sex, body mass index, family structures and smoking habits

**A linear trend was tested by assigning ordinal values for fracture frequency groups

## Discussion

This study comprehensively examined the relationship between fractures and health status in independent older adults based on a survey of community-dwelling residents. As of 2024, the proportion of individuals aged 65 and older is around 29% in Japan, while in Toyoake City, it is around 26%, showing no substantial difference [24, 25]. Although the response rate for the survey was not exceptionally high, it was > 70%, suggesting that the data reasonably reflects the situation in Japan. Among the population surveyed, 15% had experienced a fracture after the age of 65. Although this study is cross-sectional and cannot establish causality, the results indicate that fractures are associated with multiple aspects of health status, including ADL, physical function, mental health, and cognitive function. These associations suggest that past fractures may contribute to health deterioration in independent older adults.

With regard to questions on physical function, ADL, IADL, depression, and memory, the results showed that as the number of fractures increased, functional status significantly worsened across all assessed domains. Therefore, our findings support the hypothesis that fragility fractures influence depression and memory as well as physical function and IADL. The mechanism by which fragility fractures can affect mental health and cognitive abilities may involve a decline in physical function and ADL, which could exacerbate these issues [12, 26]. In the medical conditions, depression also exhibited a notable relationship with fractures, further highlighting a strong correlation between the two. Respiratory diseases also showed an association with fractures. Both respiratory diseases and fragility fractures significantly reduced activity levels in older adults, suggesting an interplay that healthcare providers must consider [27]. In addition, a strong association between multimorbidity and fragility fractures has been previously reported, and our findings align with these observations [28, 29].

Regarding subjective geriatric complaints, this study underscores the importance of acknowledging their association with fractures, even in independent older adults. Among these, dysphagia showed the strongest association with fractures. Previous study has indicated an association between kyphotic deformity of the spine, which is caused by fragility vertebral fractures, and dysphagia [30]. Therefore, the data must be analyzed with these factors in mind. Dysphagia in older adults can lead to malnutrition over time and is a potential cause of future aspiration pneumonia [31]. Therefore, emphasis should be given to dysphagia in independent older adults with fractures. To manage complaints significantly associated with fractures, including dysphagia, medications targeting these conditions can sometimes be recommended. However, it is equally important to exercise caution regarding the prescription of potentially inappropriate medications for older adults [32]. This is crucial because some medications can increase the risk of future fragility fractures [33]. Furthermore, polypharmacy, which involves the use of multiple medications, can further increase the risk of falls and fragility fractures [34]. To prevent recurrent fractures in this population, careful medication management is essential.

Taken together, this study confirmed that fractures are associated with various aspects of health status in independent older adults. Therefore, ensuring the establishment of a community-wide system that promotes regular bone density assessments is crucial for the early diagnosis of osteoporosis and fragility fracture prevention in older adults. Implementing community-based exercise programs aimed at strengthening muscles and improving balance could be an effective strategy for fall prevention. Improving safety by eliminating steps and installing nonslip flooring in homes and public facilities, etc., would help reduce fragility fractures in older adults in community dwellings.

This study has several limitations. First, although data were obtained from a survey of residents in a representative suburb of Japan, the findings are not generalizable to community-dwelling populations in other countries. However, because Japan leads the world in longevity [35], these findings may serve as a valuable reference for countries in which proportion of older adults is expected to increase. Second, this survey was not designed with a focus on fractures. For example, the survey did not include questions regarding the use of osteoporosis treatments, leaving the current status of osteoporosis management in independent older adults unclear. Furthermore, the questions related to fractures in the questionnaires have not undergone validation. Although the analysis allowed for the identification of certain characteristics of independent older adults with fractures, it did not provide a detailed understanding of these features. Third, preventive measures were taken against COVID-19 during the study period in Japan. Since this survey was conducted via mail, these measures were unlikely to have affected the survey itself. However, as several questions were related to social participation, the responses may have been underestimated compared to usual conditions. Fourth, self-rated health status and self-reported disease information may be subject to bias due to cognitive decline. This bias is not limited to cases with apparent dementia but may also arise from mild cognitive impairment, which is commonly observed in older adults. Even mild cognitive decline can affect the ability to provide responses that objectively reflect one’s health status. Therefore, this study must also consider these potential biases. Fifth, because we performed the same statistical tests multiple times, our results might contain chance findings due to multiple comparisons. In this cross-sectional study, we intended to pick up several factors associated with fractures widely. By using the follow-up cohort data, we will clarify what adverse health effects fractures could have in the older population. Nevertheless, this study utilized a relatively large and highly representative sample (response rate of 71.6%) of community-dwelling residents to identify potential factors associated with fractures among independent older adults.

In conclusion, a decline in ADL, physical function, mental health, and memory were significantly associated with fractures among independent older adults in Japan. Respiratory diseases, depression, and ophthalmologic diseases were significantly associated with fractures. Several subjective geriatric complaints were significantly associated with fractures, with dysphagia showing the strongest association.

## Data Availability

The datasets generated and/or analyzed during the current study are not publicly available due to the limitations of ethical approval involving patient data and anonymity; however, they are available from the corresponding author on reasonable request.

## Abbreviations

ADL: Activities of daily living
BMI: Body mass index
IADL: Instrumental activities of daily living
